# Developing and Evaluating a Continuous Education Program for Healthcare Assistants in Macao: A Cluster-Randomized Trial

**DOI:** 10.3390/ijerph18094990

**Published:** 2021-05-08

**Authors:** Pak-Leng Cheong, Nanly Hsu

**Affiliations:** 1 Education Department, Kiang Wu Nursing College of Macau, Macau 999078, China; 2 Faculty of Medicine, Macau University of Science and Technology, Macau 999078, China; nanlyhsu@gmail.com

**Keywords:** healthcare assistant, continuous education, competence

## Abstract

The shortage of healthcare human resources is an important challenge for coping with the aging society in Macao. Since little attention has been paid to continuous education of healthcare assistants, this study aims to develop and evaluate a continuous education program, supporting the expansion and optimization of the competence of healthcare assistants. It is a cluster-randomized trial study. All healthcare assistants who were employed in nursing homes in Macao were eligible for this study. Six nursing homes were recruited and randomly assigned either an experimental group (3 nursing homes; 45 healthcare assistants) or a control group (3 nursing homes; 40 healthcare assistants). Healthcare assistants were assessed at baseline and after intervention with the Healthcare Assistants Care Knowledge Test and the Healthcare Assistants Care Competence Self-Assessment. The experimental group received a continuous education program with 10 themes during 2017–2018 while the control groups did not. The results of the generalized estimating equation showed that care knowledge in the experimental group was significantly different from that of the control group (Wald Chi Square = 3.848, *p* < 0.05) as well as care competence (Wald Chi Square = 13.361, *p* < 0.001). This study developed a continuous program for health assistants and provided evidence that continuous education programs improve and maintain the level of care knowledge and care competency of healthcare assistants.

## 1. Introduction

Facing an aging population and growth of chronic diseases, the demand for care is increasing with a shortage of health human resources; multiple-levels of the World Health Organization reported that a Skill Mix model has been widely adopted throughout many healthcare institutions [1]. Skill Mix is an organizational unit that combines individuals of different positions and occupations according to skills or activity needs [2]. Utilizing one of the nursing skill mix models as an example, professional health workers appoint and transfer jobs to non-professional healthcare workers to partially assist with care. This method can expand the accessibility of health services for the region, but it cannot improve health in a short period [3]. Registered nurses are responsible for assigning the appropriate nursing tasks to health auxiliaries, which should be based on the characteristics and complexity of care needs, limited nursing knowledge, skills, education, and experience; practical scope of health auxiliaries should also be considered [4,5]. However, a large audit of health auxiliary personnel found that the competence of health auxiliary personnel was not fully utilized and some of the main reasons included limited training and inadequate practice assignment [5]. Therefore, the competence and continuous education of health auxiliary personnel could not be ignored.

### 1.1. Literature Review

#### 1.1.1. Overview of Health Auxiliary Training

Various terms and definitions exist for health auxiliaries. Auxiliary nurse is one term that represents individuals who have undergone nurse training or served as a nursing apprentice; other terms also exist such as nurse assistant, healthcare assistant. Auxiliary nurses have basic nursing skills but have not received formal training in the nursing decision-making process [3]. Training for health auxiliaries varies throughout the world. For example, the US federal legislation requests nurse assistants complete a license renewal every year, which includes completion of at least 12 h of in-service training and continuous learning per year, to meet the healthcare needs of residents [6]. Australia has a similar requirement for healthcare assistants in which individuals receive continuing education and study leave when they enter into nursing care [7]. Continuous education has been proven to increase the confidence and competence of health practitioners [8], providing healthcare personnel with up to date knowledge and skills and ultimately enhancing health services to vast populations [9]. Health auxiliary personnel play an essential role and function within the nursing team. As health auxiliaries are semi-professional and formal caregivers, continuing education is important. Providing ways to learn and update knowledge and skills ultimately helps to maintain and improve the quality of care [10].

Studies surrounding continuing education for health auxiliary personnel have shown many positive effects, and most of them were linked to improvement in working conditions and career recognition [11,12]. Regarding the themes of the related training, most are independent theme-designed training programs, such as preventing abuse training [13], rehabilitation care program [14], and palliative care training [15]. Using self-evaluation, mutual-evaluation, knowledge tests, and other methods to evaluate training outcomes, the results highlighted that there are diverse topics and evaluations for the training of nurse assistants. However, other than non-systematic program and single thematic training, very little literature was found regarding an integral continuous educational plan for on-the-job health auxiliary.

In regards to the methods of training, a systematic review pointed out that for healthcare workers, the learning outcomes of traditional didactic methods are not satisfactory. Case-based, clinical simulation, practice, and response methods are considered to be more effective methods of education [16]. Studies also showed that the more involved healthcare personnel were in the selection of learning resources, the more effective learning outcomes will be, leading to stable knowledge improvement [17]. Additionally, care skills and performance are more likely to be improved if training is provided in the workplace or a similar environment; printed materials are the preferred method for on-site training with repeated intervention being more beneficial to learning outcome than that of singular intervention [16].

#### 1.1.2. Healthcare Assistants in Macao

It is predicted that the elderly population of Macao will rise 19.9% by 2036 [18], indicating that Macao is gradually entering into an aging society and a super-aging society. It is expected that there will be a high demand for elderly services in the future. At this time, Macao is facing a shortage of nursing manpower as the ratio of nurses per thousand people in Macao is 3.7 [19], which is lower than the average ratio of 8.8 per thousand set by the Organization for Economic Co-operation and Development [20]. However, in response to the shortage of nursing manpower, nursing homes in Macao have been applying a skill mix model for many years by adding auxiliary staff to the nursing care team, which helps to assist registered nurses in daily nursing care. One of the main health auxiliary personnel roles is “healthcare assistant”, previously called “nurse assistant”. The minimum entry requirement for a healthcare assistant in a nursing home was completion of two or more years of nursing care training and the capability to carry out specific care independently with some tasks delegated or supervised by registered nurses [21]. In regards to continuing education for healthcare assistants in Macao, there are limited continuous education opportunities and career pathway development options; therefore, the purpose of this study is to develop and evaluate a continuous education program for healthcare assistants based on their care knowledge and competence. This is the first time an integrally designed continuous education program for healthcare assistants will exist, providing evidence for maintaining and extending nursing human resources.

## 2. Materials and Methods

The study was a cluster-randomized trial design, with the nursing homes defined as the clusters. A cluster-randomized trial design was used because it is the most feasible method and it prevented contamination between the experimental and control groups. The study was reported in accordance with the CONSORT checklist.

### 2.1. Participants

The target subject of this study was healthcare assistants who were employed in nursing homes in Macao. The eligible criteria of participated units included: (1) the nursing home was a residential institution for the elderly and (2) the institution was under the supervision and subvention of the Macao Special Administrative Region government. According to the Bureau of Social Work in Macao, there were 10 residential nursing homes funded by the Macao Special Administrative Region government; all of them were the eligible clusters in this study. The eligibility criteria of participants included: (1) employed by an eligible nursing home as a full-time healthcare assistant and (2) good Chinese language proficiency. Exclusion criteria included: (1) currently participating in other relevant training programs and (2) responsible for non-nursing care work. A total of 104 eligible healthcare assistants worked in the selected nursing homes. Allocation was based on the clusters and all healthcare assistants at a nursing home received the same intervention and were aware of their assignment after pre-test.

### 2.2. Continuous Education Program

Competency standards provide guidance on the continuous education program so that the outcome of the continuous education program corresponds to the occupational competence requirements, ensuring the efficiency of human resource training and development [22]. Regarding the competency standards of a healthcare assistant in Macao, Cheong and Hsu [23] reported a framework of competency standards, which based on “The practice and duty of registered nurse, healthcare assistant and personal caregiver” from Social Welfare Bureau of Macao [21], synthesized relevant literature and competency standards from other adjacent areas. An expert panel was established, including consultants and managers from the nursing home, clinical nurses, and educators, to ensure the competency standards were appropriate in practice. The care competence standards of healthcare assistants was developed, which covered six domains and consisted of 69 itemized care competencies.

The continuous education program was designed according to the care competence of healthcare assistants as shown in Table 1. The teaching content referred to the care certificate course in England [24], the healthcare professional service manual of Hong Kong residential nursing home [25], and healthcare assistant courses in Macao [26]. Consequently, 10 care teaching themes with teaching outlines were developed according to the care competence of healthcare assistants. The program handbook with 10 chapters was also produced, which was a guide to assist healthcare assistants with implementing the assignment, case study, and discussions. The teaching plan and learning materials were jointly discussed and formulated by a researcher who is also a nursing educator, with two nursing managers and two senior nurses in the nursing homes, as shown in Table 1. To optimize learning resources and create learning opportunities, a series of relevant books were distributed to healthcare assistants in each experimental group. All teaching materials were designed in Chinese.

A researcher and senior nurses in the nursing homes facilitated the course. On the one hand, the program can maintain consistency and reliability, on the other hand, the teaching content can be customized to the workplace. The course schedule was developed after consultation with each nursing home; the program was held at each nursing home. The course was held in person, based on a one theme per hour format, for a total program duration of about 4–6 months. The course was mainly held in the activity room or conference room of each nursing home and class time was planned between two working shifts to allow the maximum number of healthcare assistants to participate. To ensure every healthcare assistant received all course content, nursing homes with a large number of healthcare assistants were provided with a second course round for those who could not attend the first round. Data was collected before and after the course in each nursing homes between December 2017 and June 2018.

### 2.3. Instruments

Data collection instruments were demographic data, healthcare assistants care knowledge test, and healthcare assistants care competence self-assessment. Printed questionnaires for healthcare assistants in both groups were administered before intervention (pre-test) and after the program (post-test). The details are as follows.

#### 2.3.1. Demographic Data

Based on previous research, demographic information included personal and educational factors: age, education level, working years in a nursing home, other health service working experience, overall dependency of residents from the nursing home, and in-service training in the last year.

#### 2.3.2. Healthcare Assistants Care Knowledge Test

To evaluate the outcome of the continuous education program, a care knowledge test with 20 multiple-choice questions was designed, according to the care competence of healthcare assistants and the teaching contents of the course. Questions in the care knowledge test were identified as either very important or extremely important based on the knowledge list of the national care committee of the United States [27]. After the questionnaire design was completed, an expert panel was consulted and their comments were further discussed to adjust the final care knowledge test. Regarding scoring of the care knowledge test, one mark would be given when a question was answered correctly, no mark would be given for an incorrect answer.

#### 2.3.3. Healthcare Assistants Care Competence Self-Assessment

This self-assessment form was developed according to care competency standards, which was designed with the Likert 5-point option, assessing the healthcare assistants’ individual level of confidence in the following care competencies: 5, being fully confident; 4, being most confident; 3, being half confident; 2, little confident; and 1, being not confident. The content validity index (CVI), carried out through expert consultation, was 0.97. Internal consistency analysis was also conducted; Cronbach alpha was 0.82–0.96, indicating that the scale had good reliability and validity. Furthermore, it is believed that self-assessment of care competence is not only an instrument to measure what individuals recognize about their ability, but also provides a learning tool which helps individuals reflect on their current practice, and thus, may trigger the motivation of self-directed learning [28].

### 2.4. Sample Size

G-Power 3.1 (University of Düsseldorf, Düsseldorf, Germany) was used for sample estimation, the effect size set as low = 0.15, with Alpha = 0.05 and power (1-ß) = 0.8. A total of 31 participates were needed in each group. The target number of participants was increased to 35 in each group to allow for the 10% dropout rate. Since the population of this study was small, therefore, all healthcare assistants from available nursing homes were recruited as potential participants.

### 2.5. Data Analysis

SPSS 22.0 (IBM SPSS Statistics, Armonk, NY, USA) was used for data analysis. Data were rechecked for errors after being imported. Firstly, participants’ characteristics were summarized and tested for differences between the two groups by using chi-square. Descriptive statistics were analyzed to describe mean, standard deviation (SD), and the differences between pre-test and post-test in two groups. For inferential statistics, to assess the effect of the program, a generalized estimating equation (GEE) was applied to approach the repeated measurements in regression analyses; which also dealt with the potential clustering effect [29]. The alpha for significance was set at 0.05.

### 2.6. Ethics Consideration

This study was approved by the Macau University of Science and Technology (MUST/16/077/FH-E). Study permission was obtained from all participants within the nursing homes. Written informed consents were given and obtained by all health care assistants that participated and health care assistants were informed about their right to withdraw. All records are anonymous and only accessed by the members of the research team.

## 3. Results

**Figure 1:**
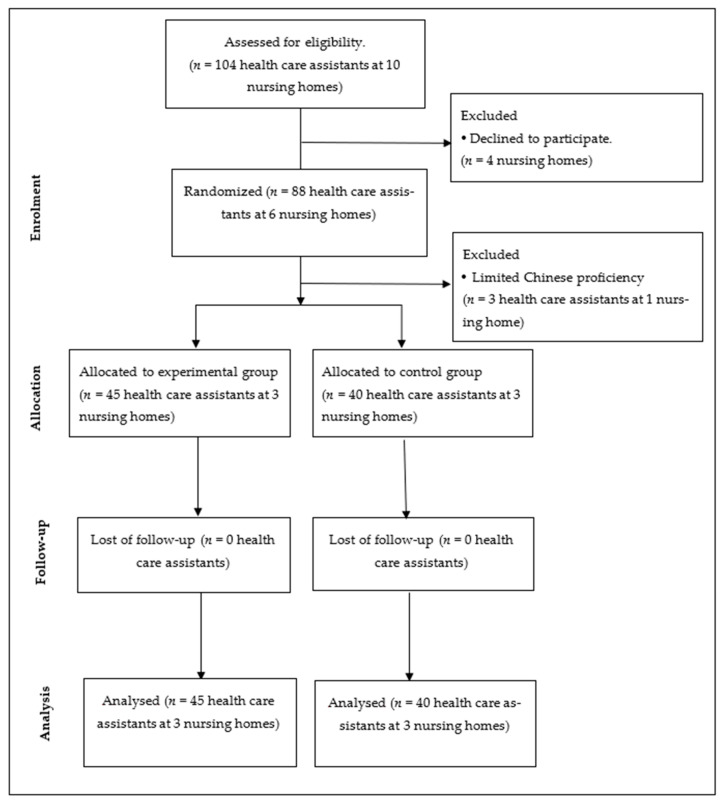
Sampling and flow of subjects.

Of the ten nursing homes, four nursing homes declined to participate in the study; the remaining six nursing homes agreed to participate in the study. The six nursing homes were randomly assigned by using random number generation in Microsoft Excel (Microsoft Corporation, Redmond, WA, USA) to either the experimental group or the control group. All healthcare assistants in the six nursing homes agreed to participate and the flow of participants at each stage are shown in Figure 1. Before allocation, three healthcare assistants were excluded because they were not proficient in Chinese. Finally, the analysis included 45 healthcare assistants in the experimental group and 40 healthcare assistants in the control group. In accordance with the teaching plan, the continuous education program had been executed in each nursing home of the experimental group after the pre-test was completed, while the control groups did not receive this program after pre-test completion. After completing the course, the post-test data of both groups was collected at the same time.

### 3.1. Demographic Data

Among the 85 healthcare assistants, all of them were female with a majority age under 35 years (77.6%) and have worked in a nursing home for 3 years or less (67%). The distribution of other demographic data is shown in Table 2. The chi-square test found that most of the basic data showed no significant differences between the two groups, except the data related to “age, working years in a nursing home, in-service training in last year”, which were significantly different. Therefore, on the analysis of the effect of intervention, the “age, working years in a nursing home, in-service training in last year” were set as covariance when comparing the program intervention effect.

### 3.2. Descriptive Statistics

In terms of care knowledge, after receiving the course, all six domains of care knowledge in the experimental group were increased, with a difference of 0.09–0.46 between the pre-test and post-test scores. On the other hand, the difference between before and after care knowledge of the control group was −0.12–0.34, the negative changes are shown in “Clinical care skills”, “Healthcare promotion”, and “Psychosocial support”, as shown in Table 3. In the field of care competence, within the overall average score of care competencies of the experimental group, post-test result was better than the pre-test. All six domains were changed positively, with a difference of 0.02–0.29 between the pre-test and post-test scores. Contrarily, the difference between before and after self-assessment of the control group was −0.04–0.13, which is noted in the negative change in “Clinical care skills”, as shown in Table 3.

### 3.3. Inferential Statistics

Care knowledge and care competence of the experimental and control groups were analyzed by GEE. Independent variables in GEE analyses included: group (experimental or control); time (pre-test or post-test); and interaction of group and time; the dependent variable was care knowledge and care competence. After controlling the variables of age, working years in nursing home, and in-service training in the last year, the results of the GEE showed the care knowledge in the experimental group was significantly different from that of the control group (Wald Chi Square = 3.848, *p* < 0.05), as well as the care competence (Wald Chi Square = 13.361, *p* < 0.001) (see Table 4). The result shows that the continuous education program had a significantly positive outcome on the care knowledge and care competence of healthcare assistants.

## 4. Discussion

The study results showed that healthcare assistants who were currently working in nursing homes and using care knowledge and skills in daily work were at confident level of overall care competence. This result was similar to the result of Tsai [30], a study on the caring ability of nurse-aides in long term care facilities in Taiwan. The overall results showed the nurse-aides to be at the level of mostly or fully mastered. Regarding the clinical role of healthcare assistants, the care competence “Taking the role of healthcare assistants” is the most obvious improvement in the experimental group. This suggested the need, which was met by the outcome of the course, for healthcare assistants to promote the recognition and collaboration on the role of the care team in the nursing homes; this result confirms the previous evidence [31].

The course also provided a platform for healthcare assistants to discuss ways to improve quality of care. Care knowledge in “Daily living care” after the program was improved significantly, but self-assessment of care competence showed the lowest improvement. The progression from knowledge to competence is a complex pedagogical process. This study revealed that improvement of knowledge may be insufficient to empower healthcare assistants’ confidence in the workplace; similar findings are reported in previous studies [15], which raises further questions to research.

“Clinical care skills” accounts for the most care competence items, which was the best care competence among the six domains in the pre-test. Most of the healthcare assistants have received systematic training in clinical care skills according to their educational background. Therefore, healthcare assistants were most confident in “Clinical care skills”. The course in this study provided skill maintenance and updated relevant information. Although the care knowledge and care competence of healthcare assistants in the “Clinical care skills” category showed a little improvement after the course, the control group regressed in care knowledge and competence in the post-test. It is suggested that the continuous education program maintained the level of knowledge and competence. On the other hand, “Healthcare promotion” refers to assisting different rehabilitation training and self-care skills, whereas residents’ needs vary based on their dependency, for example, highly dependent residents require clinical care and daily living care. It can be seen from the demographic data that the majority of healthcare assistants were taking care of residents with a high degree of dependence. As a result, the course provided knowledge of healthcare promotion of healthcare assistants, but they may not have much confidence in their healthcare promotion due to lack of practical implementation.

This is the first study specifically designed for the continuous education program of healthcare assistants in nursing homes in Macao. The study provided evidence that continuous education improved and maintained the level of knowledge and competence. Different from past care training courses, it was a multi-themed continuous education program, jointly designed and implemented by nursing educators and clinical nurses. We believe that healthcare assistants can benefit greatly from learning and sharing experiences in the workplace through discussion with mentors, which has been pointed out by Bluestone et al. [16]. We suggest that nursing homes regularly organize continuous education programs according to the healthcare assistants care competence framework to provide appropriate learning resources and learning activities in the workplace; therefore, improving care competence, safety, and quality care service for the elderly.

Due to the limited time period, this study was unable to investigate further impacts of the program on care knowledge and care competence. It is suggested that long-term studies should be conducted in the future to continuously evaluate healthcare workers in order to verify the long-term effect of the program. Furthermore, the researcher in this study was the main mentor within the continuous education program. Ideally, the most desirable mentor is an educator, nurse, and manager in the nursing home. Therefore, it is suggested to study and establish a mentor course for educators, nurses, and managers in nursing homes in the future.

This study relied on the coordination and cooperation of various nursing homes, as such, the teaching content and evaluation methods were limited. It is suggested that nurses and mangers can explore further opportunities for inter-professional interaction, such as allowing healthcare assistants to participate in multi-disciplinary case conferences and residents’ meetings. Allowing healthcare assistants to fully understand their clients is a way to enhance learning and promote care competencies and care services. The more healthcare assistants understand their clients, the more secure and effective they are in their care work. Moreover, the knowledge test and self-assessment were chosen as a way to evaluate the care knowledge and care competence of healthcare assistants, as an objective and a subjective measure, since there were limits to the application of other measures due to the numerous differences amongst the six nursing homes. Therefore, it is suggested that multiple methods be applied to evaluate the care competence of healthcare assistants within a workplace, such as performance in daily work, feedback from colleagues and residents, and skills test, in order to comprehensively understand the specific competency level of healthcare assistants.

## 5. Conclusions

In the report of the global health workforce, the World Health Organization proposed “no health without a workforce” [1]. In the plight of the global health workforce shortage, it is vital to reasonably allocate the tasks of healthcare workers and optimize the competence of healthcare professionals. In this study, according to healthcare assistants’ care competence, the continuous education program was designed to provide a learning platform for healthcare assistants in the nursing home. The continuous education program in this study differs from prior studies. It has multiple teaching themes, rather than an independent theme design of training, to explore the overall status and evaluate the six domains of care competence. While the continuous education and career development of healthcare assistants are unclear in Macao, this study developed a continuous program for health assistants and found that the care knowledge and care competence of healthcare assistants were maintained and improved through a continuous education program, providing a significant pilot to support the development of nursing human resources.

## Figures and Tables

**Table 1:** The teaching plan of the continuous education program.

Themes	Teaching Content	Teaching Methods
1. Role and Task	- Meaning of care - Role of health care assistant - Team and cooperation	Lecture, case sharing
2. Caring and Respect	- Principle of privacy - Religion and respect - Decision-making support	Lecture, discussion
3. Emergency Event	- CPR - Reporting - Responding to emergency event	Lecture, demonstration, and practice
4. Safety	- Prevention of fall and pressure sores - Wound care	Lecture, discussion, case sharing
5. Hygiene	- Meaning of comfort - Personal hygiene	Experiential learning
6. Infection Control	- Controlling infective diseases - Hand hygiene	Lecture, demonstration, and practice
7. Nutrition and Elimination	- The need of water and nutrients - Assist with feeding and elimination	Lecture, discussion
8. Activities and Rest	- Assist with turn /walk/ exercise - Ensure sleep quality	Lecture, demonstration, and practice
9. Symptom Control	- Assessment of pain and fever - Health promotion of chronic diseases	Lecture, case studies
10. Special Care	- Palliative care - Communication skill for dementia and emotional residents	Lecture, case sharing

**Table 2:** Demographic data.

Variable	Items	Experimental Group (*n* = 45)	Control Group (*n* = 40)	*p*
		Number (%)	Number (%)	
Age	25 years or below	23 (51.1)	6 (15.0)	0.002 **
	26–35 years	15 (33.3)	22 (55.0)	
	36 years or above	7 (15.6)	12 (30.0)	
Educational level	Secondary	11 (24.4)	8 (20.0)	0.116
	Diploma	30 (66.7)	21 (52.5)	
	Bachelor	4 (8.9)	11 (27.5)	
Working years in a nursing home	Less than 1 year	10 (22.2)	9 (22.5)	0.002 **
	1–3 years	28 (62.2)	10 (25.0)	
	4–7 years	4 (8.9)	12 (30.0)	
	More than 7 years	3 (6.7)	9 (22.5)	
Other health service working experience	No	7 (15.6)	4 (10.0)	0.529
	Yes	38 (84.4)	36 (90.0)	
Overall dependency of residents from the nursing home	High dependency	33 (73.3)	35 (87.5)	0.173
	Middle dependency	12 (26.7)	5 (12.5)	
In-service training in last year	No	6 (13.3)	13 (32.5)	0.040 *
	Yes	39 (86.7)	27 (67.5)	

* *p*-value < 0.05; ** *p*-value < 0.01.

**Table 3:** Means and standard deviations of care knowledge and care competence.

Instruments	Domains	Experimental Group (*n* = 45)			Control Group (*n* = 40)		
		Pre (M ± SD)	Post (M ± SD)	Difference	Pre (M ± SD)	Post (M ± SD)	Difference
Care knowledge	Daily living care	0.55 ± 0.20	0.76 ± 0.21	0.21	0.50 ± 0.23	0.50 ± 0.25	0.00
	Clinical care skills	0.58 ± 0.19	0.64 ± 0.15	0.06	0.49 ± 0.18	0.45 ± 0.14	−0.04
	Healthcare promotion	0.58 ± 0.30	0.67 ± 0.32	0.09	0.63 ± 0.27	0.51 ± 0.33	−0.12
	Psychosocial support	0.73 ± 0.29	0.88 ± 0.22	0.15	0.66 ± 0.32	0.56 ± 0.34	−0.10
	Residents’ rights and interests	0.56 ± 0.39	0.73 ± 0.36	0.17	0.44 ± 0.36	0.51 ± 0.37	0.07
	Taking the role of health care assistants	0.40 ± 0.27	0.86 ± 0.25	0.46	0.35 ± 0.28	0.69 ± 0.27	0.34
	Overall care knowledge	0.57 ± 0.15	0.72 ± 0.12	0.15	0.51 ± 0.13	0.51 ± 0.14	0.00
Care competence	Daily living care	4.45 ± 0.49	4.47 ± 0.49	0.02	4.18 ± 0.44	4.18 ± 0.41	0.00
	Clinical care skills	4.52 ± 0.34	4.58 ± 0.34	0.06	4.20 ± 0.43	4.15 ± 0.50	−0.04
	Healthcare promotion	3.84 ± 0.79	4.02 ± 0.68	0.18	3.71 ± 0.71	3.78 ± 0.67	0.07
	Psychosocial support	4.21 ± 0.54	4.43 ± 0.42	0.22	4.04 ± 0.47	4.08 ± 0.49	0.05
	Residents’ rights and interests	4.48 ± 0.48	4.67 ± 0.34	0.19	4.23 ± 0.53	4.35 ± 0.54	0.13
	Taking the role of health care assistants	3.95 ± 0.77	4.24 ± 0.58	0.29	3.74 ± 0.71	3.83 ± 0.58	0.09
	Overall care competence	4.37 ± 0.38	4.48 ± 0.36	0.11	4.10 ± 0.41	4.11 ± 0.45	0.01

**Table 4:** Generalized estimating equation (GEE) analysis of care knowledge and care competence (*n* = 85).

Variable	Parameters	β	SE	Wald χ^2^	95% CI	*p*
Care knowledge	Intercept	0.410	0.036	127.28	(0.339, 0.482)	<0.001 ***
	Group (Experimental vs. Control)	0.057	0.029	3.848	(0.000, 0.114)	0.049 *
	Time (Post-test vs. Pre-test)	0.003	0.019	0.021	(−0.034, 0.040)	0.885
	Group X Time	0.149	0.032	21.138	(0.086, 0.213)	<0.001 ***
Care competence	Intercept	4.077	0.162	633.099	(3.759, 4.394)	<0.001 ***
	Group (Experimental vs. Control)	0.349	0.095	13.361	(−0.37, 0.238)	<0.001 ***
	Time (Post-test vs. Pre-test)	0.006	0.439	0.017	(−0.080, 0.092)	0.897
	Group X Time	0.100	0.070	2.045	(−0.037, 0.238)	0.153

* *p*-value < 0.05; *** *p*-value < 0.001.

## Data Availability

All data that support the findings of this study are available from the corresponding author upon reasonable request.
